# Circular RNA circNTRK2 facilitates the progression of esophageal squamous cell carcinoma through up-regulating NRIP1 expression via miR-140-3p

**DOI:** 10.1186/s13046-020-01640-9

**Published:** 2020-07-11

**Authors:** Xiaoqi Chen, Jing Jiang, Yunxia Zhao, Xinting Wang, Chuanlei Zhang, Lv Zhuan, Danyang Zhang, Yuling Zheng

**Affiliations:** 1 Department of Gastroenterology, The First Affiliated Hospital of Henan University of Chinese Medicine, No. 19 Renmin Road, Jinshui District, Zhengzhou, 450000 China; 2 School of Mechanical and Power Engineering, Zhengzhou University, Zhengzhou, 450000 China; 3 Henan University of Chinese Medicine, Zhengzhou, 450000 China; 4 Department of Medical Administration, The First Affiliated Hospital of Henan University of Chinese Medicine, Zhengzhou, 450000 China; 5 Department of Pharmacy, Zhengzhou Hospital of Traditional Chinese Medicine, Zhengzhou, 450000 China; 6 Guoyitang, The First Affiliated Hospital of Henan University of Chinese Medicine, Zhengzhou, 450000 China

**Keywords:** Esophageal squamous cell carcinoma, circNTRK2, miR-140-3p, Nuclear receptor-interacting protein 1

## Abstract

**Background:**

Esophageal squamous cell carcinoma (ESCC) is one of the most prevalent gastrointestinal malignancies with high mortality. Circular RNAs (CircRNAs) have become a research hotspot in recent years for their vital roles in cancer development and progression. This study aims to clarify the roles of circNTRK2 and its underlying molecular mechanisms in ESCC.

**Methods:**

The levels of circNTRK2, miR-140-3p, and nuclear receptor-interacting protein 1 (NRIP1) mRNA were examined by qRT-PCR. The cell proliferation ability was detected via CCK-8, EdU and colony formation assays. The invasion capacity was tested by using transwell assay. The apoptotic rate was evaluated through flow cytometry. The protein levels of cleaved PARP, cleaved caspase-3, E-cadherin, vimentin, and NRIP1 were measured by western blot assay. The validation of circular structure was performed by Sanger sequencing, divergent primer PCR, and RNase R treatments. The ceRNA regulatory mechanism of circNTRK2 was observed via dual-luciferase reporter, RIP and RNA pull-down assays. The mice xenograft models were constructed to confirm the oncogenicity of circNTRK2 in ESCC in vivo.

**Results:**

CircNTRK2 was highly expressed in ESCC tissues and cells. High expression of circNTRK2 was correlated with advanced TNM stage, lymph node metastasis and short survival. Knockdown of circNTRK2 inhibited ESCC cell proliferation, invasion and epithelial-mesenchymal transition (EMT), and accelerated apoptosis in vitro. Mechanistic assays disclosed that circNTRK2 could act as a sponge for miR-140-3p to abate its suppression on target NRIP1 expression. Moreover, miR-140-3p-induced inhibitory effects on ESCC cell malignant phenotypes were attenuated by the overexpression of circNTRK2. In addition, depletion of NRIP1 impeded cell proliferation, invasion and EMT, while enhanced apoptosis. Furthermore, silencing of circNTRK2 suppressed cell proliferation and invasion through regulating NRIP1 expression. Also, knockdown of circNTRK2 slowed ESCC tumor growth in vivo.

**Conclusion:**

CircNTRK2 promoted ESCC progression by regulating miR-140-3p/NRIP1 pathway. Our findings contribute to a better understanding of circRNAs as miRNA sponges and highlight a promising therapy target in ESCC.

## Background

Esophageal carcinoma ranks ninth in the new diagnosed cancer cases and occupies sixth in cancer deaths worldwide [1]. Esophageal squamous cell carcinoma (ESCC), the most common histological subtype of esophageal carcinoma, accounts for approximately 90% of all cases globally [2]. ESCC is highly prevalent in East Asia, South/East Africa and South Europe [3, 4]. Multimodal treatment encompassing surgery, radiation and chemotherapy is currently the main therapeutic option for ESCC [5]. Due to the lack of early clinical symptoms, the majority of ESCC patients are diagnosed at the advanced stages. The prognosis for esophageal carcinoma is poor, and the 5-year relative survival rate of patients with distant metastasis is only 5% [6]. Therefore, identifying novel biomarkers and molecular targets is urgently needed for improving the outcomes of ESCC patients.

Circular RNAs (circRNAs), a class of non-coding transcripts generated by pre-mRNA back splicing, is characterized by a covalently closed loop without 5′ caps and 3′ tails [7]. CircRNAs have drawn increasing attentions for their important participation in the genesis and development of human cancers at transcriptional, post-transcriptional, and translational levels [8]. CircRNAs could act as miRNA sponges to affect the biological activity and function of their target mRNAs [9]. For example, circARHGAP10 accelerated cell proliferation and migration in non-small-cell lung cancer by targeting the miR-150-5p/GLUT1 axis [10]. Hsa_circ_0068871 facilitated bladder cancer progression via up-regulating FGFR3 expression and activating STAT3 signaling by serving as a sponge of miR-181a-5p [11]. In recent years, several circRNAs such as ciRS-7 [12], circPRKCI [13] and circGSK3β [14], have been found to be aberrantly expressed in ESCC and play important roles in cancer process. CircNTRK2 (hsa_circ_0087378), located at chr9:87356806–87,367,000 with a length of 237 bp, is formed by the circularization of 12–14 exons of Pre-NTRK2. A previous report by Yuan et al. demonstrated that hsa_circ_0087378 was down-regulated in ER-positive breast cancer, and hsa_circ_0087378/miR1260b/SFRP1 axis was proposed as a vital regulatory pathway [15]. According to the GEO database (GSE131969), circNTRK2 is identified as the most up-regulated circRNAs among all candidates. Thus, circNTRK2 was selected as a research object for further function and mechanism analysis in ESCC.

In the current study, we verified that circNTRK2 was up-regulated in ESCC tissues and cells. High circNTRIK2 was associated with TNM stage, lymph node metastasis and poor prognosis. Functionally, knockdown of circNTRK2 repressed ESCC cell proliferation, invasion and EMT in vitro, and slowed tumor growth in vivo. Mechanistically, circNTRK2 facilitated NRIP1 expression by sponging endogenous miR-140-3p. Our findings reveal a novel regulatory mechanism of circNTRK2 in ESCC and proposed a promising therapeutic target for ESCC patients.

## Materials and methods

### Patient tissue specimens

The study was permitted by the Ethics Committee of The First Affiliated Hospital of Henan University of Chinese Medicine and performed according to the Declaration of Helsinki Principles. Informed written consents were signed by each patient for using their tissues. Tumor tissue samples and adjacent normal tissues (at least 3 cm from the edge of cancer tissues) were obtained from 56 patients with a definite pathological diagnosis of ESCC. None of the participants received preoperative radiotherapy or chemotherapy. All specimens were immediately snap-frozen in liquid nitrogen and stored at − 80 °C until further used.

### Cell culture

Human ESCC cell lines (Eca-109, EC-9706, KYSE-30, KYSE-150, TE-1) were purchased from Cell Bank of the Chinese Academy of Sciences (Shanghai, China). Human normal esophageal epithelial cell line Het-1A was obtained from American Type Culture Collection (ATCC; Manassas, VA, USA). ESCC cells were cultured in RPMI 1640 medium (Invitrogen, Carlsbad, CA, USA) containing 10% fetal bovine serum and 1% penicillin/streptomycin. Het-1A cells were maintained in bronchial epithelial cell growth medium (BEGM, BulletKit, Lonza, MD). All cells were kept in a humidified incubator under an atmosphere of 5% CO_2_ at 37 °C.

### Quantitative real-time polymerase chain reaction (qRT-PCR)

Total RNA in ESCC tissues and cells was extracted using TRIzol Reagent (Invitrogen, Carlsbad, CA, USA). For circRNA and mRNA, the cDNA was synthesized by using SuperScript VILO cDNA Synthesis kit (Invitrogen; Carlsbad, CA, USA). For miRNA, reverse transcription was performed by using miRcute Plus miRNA First-Strand cDNA Synthesis Kit (Tiangen, Beijing, China). The quantitative PCR was conducted using SYBR Premix Ex Taq II (TaKaRa, Dalian, China) on a LightCycler 480 system (Roche, Basel, Switzerland). The relative gene expression was calculated by using the 2^-ΔΔCt^ method with U6 as the reference gene for miRNA and GAPDH as the internal control for circRNA and mRNA. All primers were obtained from Songon (Shanghai, China) and listed as follows:

circNTRK2: F, 5′-TCTCGGTCTATGCTGTGGTG-3′, R, 5′-CATTCGCTGCAGTTCCATAA-3′;

miR-140-3p: F, 5′-CAGTGCTGTACCACAGGGTAGA-3′, R, 5′-TATCCTTGTTCACGACTCCTTCAC-3′;

NRIP1: F, 5′-GAGCACTCCACCTTTACTTACAT-3′, R, 5′-CAATCATACCTATCGGTTTATCTG-3′;

GAPDH: F, 5′-TGTTCGTCATGGGTGTGAAC-3′, R, 5′-ATGGCATGGACTGTGGTCAT-3′;

U6: F, 5′-ATTGGAACGATACAGAGAAGATT-3′, R, 5′-GGAACGCTTCACGAATTTG-3′.

### Confirmation of circular structure

To confirm the circular structure of circNTRK2, the circular and linear transcripts of NTRK2 were amplified by divergent and convergent primers in both complementary DNA (cDNA) and genomic DNA (gDNA) from ESCC cells. Then, agarose gel was used to separate the PCR products. In theory, circNTRK2 is amplified by divergent primers in cDNA but not gDNA. Additionally, Sanger sequencing was performed to verify the sequence of circNTRK2. Meanwhile, total RNA (5 μg) extracted from ESCC cells was incubated with RNase R (3 U/μg, Epicenter, Madison, WI, USA) for 20 min at 37 °C, followed by qRT-PCR to measure the expression levels of circular and linear NTRK2.

### Cell transfection

To down-regulate circNTRK2, small interfering RNAs (siRNAs) targeting back splice junction of circNTRK2 (si-circ #1, si-circ #2, si-circ #3) were synthesized by GenePharma (Shanghai, China). NRIP1-specific siRNA (si-NRIP1) was used to silence NRIP1. Non-targeting control siRNA (si-NC) was used as a control. The sequences of siRNAs were listed as follows: si-circ #1, 5′- GCATGAAAGGTGCAAACCCAA − 3′; si-circ #2, 5′- GGCATGAAAGGTGCAAACCCA − 3′; si-circ #3, GTTTGGCATGAAAGGTGCAAA; si-NRIP1, 5′- GAGGAUCAGAACUUUAACATT-3′; si-NC, 5′-UUCUCCGAACGUGUCACGUTT-3′. MiR-140-3p mimics (miR-140-3p) and its matched control (miR-NC), miR-140-3p inhibitor (anti-miR-140-3p) and its matched control (anti-miR-NC) were obtained from GenePharma (Shanghai, China). To construct circNTRK2-overexpression plasmid (circNTRK2), the full length circNTRK2 cDNA was synthesized and then inserted into pLCDH-ciR vector (Geneseed, Guangzhou, China), while mock vector with no circNTRK2 sequence was used as a control. To establish NRIP1-overexpression plasmid (NRIP1), the coding region of NRIP1 cDNA was subcloned into pcDNA3.1 expression vector (Geneseed, Guangzhou, China). Cells were screened with puromycin (2 μg/ml) for 4 weeks to establish overexpression and control cell lines. ESCC cells were transfected with these oligonucleotides or plasmids at appropriate doses using Lipofectamine 3000 (Invitrogen, Carlsbad, CA, USA) according to the manufacturer’s manual.

### Cell counting kit-8 (CCK-8) assay

CCK-8 (Bimake, Shanghai, China) was used to evaluate ESCC cell viability. Cells (2 × 10^3^/well) were inoculated into 96-well plates. After incubation of 24 h, 48 h, 72 h, or 96 h at suitable condition, 10 μl CCK8 solution and 90 μl medium were added to each well. Two hours later, the absorbance at 450 nm was measured using a Varioskan Flash Microplate Reader (Thermo Fisher Scientific, Waltham, MA, USA).

### 5-Ethynyl-2′- deoxyuridine (EdU) assay

The proliferative ability of ESCC cells was detected by using a Cell-Light™ EdU DNA Cell Proliferation Kit (RiboBio, Guangzhou, China) following the manufacture’s guideline. Images of randomly selected fields were obtained under a fluorescence microscope (Leica, Wetzlar, Germany).

### Colony formation assay

ESCC cells were added into 6-well plates at a density of 1000 cells/well and incubated at 37 °C for half a month. After fixing with 75% ethanol and staining with 0.1% crystal violet, the colonies with more than 50 cells were counted.

### Transwell invasion assay

Transwell assay was implemented to determine the invasive capability of ESCC cells in 24-well Boyden chambers (Corning Incorporated, Corning, NY, USA) with pre-coated Matrigel (BD Biosciences, Franklin Lakes, NJ, USA). Briefly, 600 μl RPMI 1640 medium containing 10% FBS was added to the lower chamber, while 1 × 10^5^ cells in 200 μl serum-free medium was plated into the upper chamber. After incubation at 37 °C with 5% CO_2_ for 24 h, cells remaining on the upper surfaces of the transwell chambers were removed, and cells traversed to the bottom surface were fixed with 4% paraformaldehyde and stained with 0.1% crystal violet. The invasive cells were captured by a light microscope (Olympus, Japan) and counted from five randomly chosen fields.

### Flow cytometry analysis

ESCC cells (5 × 10^5^/well) were seeded into 6-well plates and cultured for 48 h at 37 °C. Then, cells were stained with an Annexin V-FITC apoptosis detection kit (BD Biosciences, San Jose, CA, USA) and subjected to a FACSCalibur flow cytometer (BD Biosciences, San Jose, CA, USA) for apoptosis detection.

### Western blot assay

Total protein from ESCC cells was extracted using RIPA lysis and extraction buffer (Thermo Fisher Scientific, Rockford, IL, USA) and quantified by BCA Protein Assay Kit (Beyotime, Shanghai, China). Subsequently, 30 μg protein samples were separated by sodium dodecyl sulfonate-polyacrylamide gel electrophoresis (SDS-PAGE) and transferred onto polyvinylidene fluoride (PVDF) membrane (GE Healthcare, Piscataway, NJ, USA). After blocking in nonfat milk overnight at 4 °C, the membranes were incubated with primary antibody against E-cadherin (1:500, Abcam, Cambridge, MA, USA), vimentin (1:1000, Abcam), Cleaved PARP (1:1000; Abcam), Cleaved caspase-3 (1:500, Abcam), NRIP1 (1:500, Abcam), and GAPDH (1:10,000, Abcam) overnight at 4 °C, and then were probed with HRP-conjugated secondary antibody for 2 h at room temprature. ECL Western Blotting Detection Kit (Solarbio, Beijing, China) was used to detect the protein bands.

### Subcellular fractionation

The RNA from nuclear and cytoplasm fractions was isolated by using a Cytoplasmic and Nuclear RNA Purification Kit (Norgen, Thorold, ON, Canada). In brief, ESCC cells were incubated with lysis solution on ice for 10 min and then centrifuged for 3 min at 12,000 g. The supernatant was collected for cytoplasmic RNA extraction and the nuclear pellet was used for nuclear RNA. qRT-PCR was used to measure the relative expression of circNTRK2 in different fractions. GAPDH was used as the cytoplasmic control, while U6 was used as the nuclear control.

### Bioinformatics analysis

The targets of circNTRK2 were predicted by CircInteractome (https://omictools.com/circinteractome-tool), CircBank (http://www.circbank.cn/) and StarBase 3.0 (http://starbase.sysu.edu.cn/) online databases. TargetScan ((http://www.targetscan.org/vert_71/), miRDB (http://mirdb.org/), DIANA-microT (http://diana.imis.athena-innovation.gr/DianaTools/index.php?r=microT_CDS/index), DIANA-TarBase (http://carolina.imis.athena-innovation.gr/diana_tools/web/index.php?r=tarbasev8%2Findex) and miRTarBase (http://mirtarbase.mbc.nctu.edu.tw/) online databases were utilized to predicted the candidate targets of miR-140-3p. GEPIA tool (http://gepia.cancer-pku.cn/detail.php?gene=&clicktag=boxplot) was used to analyze the expression of NRIP1 in cancer and normal tissues in TCGA database.

### Dual-luciferase reporter assay

To evaluate the direct binding between miR-140-3p and circNTRK2, the wild type or mutant sequences containing the binding sites of miR-140-3p in circNTRK2 were inserted into a pmirGLO vector (Promega Corporation, Madison, WI, USA), named as circNTRK2-wt and circNTRK2-mut, respectively. Subsequently, the luciferase reporter (100 ng) and miR-140-3p mimic or miR-NC (40 nM) were co-transfected into ESCC cells. After 48 h, the luciferase activity was measured using the Dual-Luciferase Reporter Assay System (Promega, Madison, WI, USA) with renilla luciferase activity as an internal reference. Similar procedures were conducted to examine the binding between miR-140-3p and NRIP1. Luciferase reporters including wild type NRIP1-wt and mutated types (NRIP1-mut1: nt 893–900, NRIP1-mut2: nt 3086–3092, and double mutation: NRIP1-mut1 + 2) were constructed.

### RNA immunoprecipitation (RIP) assay

Magna RNA immunoprecipitation kit (Millipore, Billerica, MA, USA) was used to validate the binding between circNTRK2 and miR-140-3p. Briefly, ESCC cells were washed with PBS and lysed in RIP lysis buffer. Subsequently, cell lysates were incubated with RIP buffer containing magnetic beads coupled with human anti-Argonaute 2 antibody (Ago2; Millipore) or non-specific anti-IgG (Millipore). After elution, qRT-PCR was used to determine the level of circNTRK2 in immunoprecipitated RNA.

### RNA pull-down assay

ESCC cells were transfected with the wild type biotin-labelled miR-140-3p (Bio-miR-140-3p-wt), mutant biotin-labeled miR-140-3p (Bio-miR-140-3p-mut), and non-specific negative control (Bio-miR-NC). After 48 h, cell lysates were incubated with streptavidin magnetic beads, followed by qRT-PCR to measure the level of circNTRK2 in RNA complexes.

### Mice xenograft models

All animal experiments were approved by the Institutional Animal Care and Use Committee of The First Affiliated Hospital of Henan University of Chinese Medicine and performed following the guidelines of National Institutes of Health. Male BALB/c nude mice aged 4–5 weeks were obtained from Shanghai Laboratory Animal Company (SLAC, Shanghai, China). KYSE-150 cells (5× 10^6^) stably infected with lentivirus vectors encoding shRNA against circNTRK2 (sh-circNTRK2) or a non-silencing negative control (sh-NC) were subcutaneously injected into the right armpit of nude mice (*n* = 5 per group). The length and width of xenograft tumors were measured at indicated time points, and tumor volumes were calculated according with the formula: volume (mm^3^) = width^2^ × length/2. At 25 days after inoculation, all mice were sacrificed and xenografts were dissected for further analysis.

### Statistical analysis

Data analysis was performed by using GraphPad Prism 7 (GraphPad Software, Inc., La Jolla, CA, USA). All continuous data were presented as mean ± standard deviation (SD). Differences in two groups were assessed by Student’s *t*-test, while one-way analysis of variance (ANOVA) was utilized in multiple groups. Chi-square test was applied to evaluate the relationship between circNTRK2 expression and clinicopathological parameters. Kaplan-Meier method was employed to determine the survival rate and log-rank test was used to compare the difference. Pearson’s correlation coefficients were used to detect the correlation. *P* < 0.05 was set as statistically significant.

## Results

### Validation and expression of circNTRK2 in ESCC tissues and cells

**Fig. 1:**
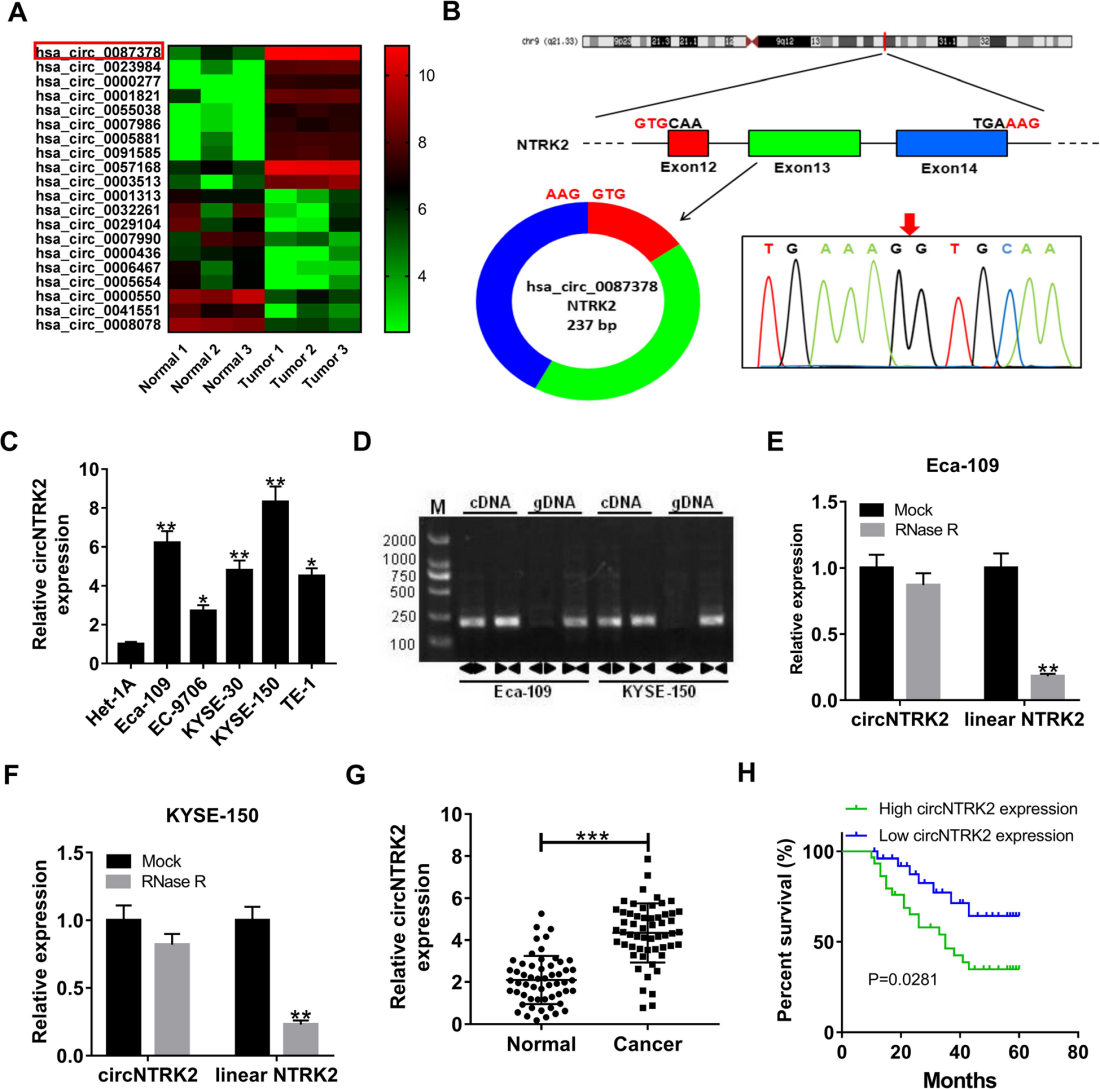
CircNTRK2 validation and expression in ESCC tissues and cells. (**a**) Heat map derived from GEO database (GSE131969) showed the top 20 differentially expressed circRNAs between ESCC samples and normal subjects. (**b**) Schematic diagram displayed that circNTRK2 was formed by the circularization of NTRK2 exon 12–14, and the “head-to-tail” splicing sites (Red arrow) of circNTRK2 was confirmed by RT-PCR and Sanger sequencing. (**c**) Expression of circNTRK2 in ESCC cell lines (Eca-109, EC-9706, KYSE-30, KYSE-150, TE-1) and normal esophageal epithelial cell line Het-1A. (**d**) The existence of circNTRK2 was validated by RT-PCR in two ESCC cell lines (Eca-109 and KYSE-150). CircNTRK2 was amplified by divergent primers in cDNA but not in gDNA. (**e** and **f**) The levels of circular and linear NTRK2 were detected by using qRT-PCR in Eca-109 and KYSE-150 cells treated with RNase R. (**g**) Expression of circNTRK2 in 56 pairs of ESCC tumor tissues and adjacent non-cancerous tissues. (**h**) The overall survival rates of ESCC patients in high or low circNTRK2 expression group was assessed by Kaplan-Meier method. *P < 0.05, **P < 0.01, ***P < 0.001

In order to seek for circRNAs aberrantly expressed in ESCC, we analyzed GEO database (GSE131969) containing 3 ESCC tissues and 3 normal tissues. Heat map exhibited the 10 most up-regulated and down-regulated circRNAs (Fig. 1A). Here, hsa_circ_0087378 was selected for further analysis due to that it is the most significantly increased circRNA. Hsa_circ_0087378 is produced from exon 12–14 of NTRK2 located on chromosome 9 and is 237 bp in length, thus we named it as circNTRK2. The back-spliced region was confirmed in the RT-PCR product of circNTRK2 by Sanger sequencing (Fig. 1B). In addition, circNTRK2 expression was significantly higher in human ESCC cell lines (Eca-109, EC-9706, KYSE-30, KYSE-150, TE-1) than that in human esophageal epithelial cell line Het-1A (Fig. 1C). Subsequently, divergent and convergent primers were designed to amplify the circular and linear transcripts of NTRK2, respectively. As shown in Fig. 1D by gel electrophoresis, circNTRK2 was amplified by divergent primers in cDNA but not gDNA, while linear NTRK2 was amplified by convergent primers in both cDNA and gDNA from Eca-109 and KYSE-150 cells. Compared to linear NTRK2 mRNA, circNTRK2 was found to be resistant to RNase R (Fig. 1E and F). Furthermore, circNTRK2 expression was significantly elevated in tumor tissue samples in contrast to that in adjacent normal tissue samples from 56 ESCC patients (Fig. 1G). Consistently, circNTRK2 expression was found to be up-regulated in 35 ESCC tumor tissues compared to adjacent non-cancerous (Supplementary Fig. 1). As shown in Table 1, high circNTRK2 expression was demonstrated to be correlated with advanced TNM stage and lymph node metastasis. Kaplan-Meier survival analysis showed that the overall survival of ESCC patients with high circNTRK2 expression were shorter in comparison with that in low circNTRK2 expression group (Fig. 1H). These data confirmed the circular structure of circNTRK2, and circNTRK2 expression was up-regulated in ESCC.

**Table 1:** Correlation between circNTRK2 expression and clinicopathological features in 56 ESCC patients

Variable	circ NTRK2 expression		*P* value
	Low (*n* = 28)	High (n = 28)	
Age			0.422
≤ 55	12	15	
> 55	16	13	
Gender			0.763
Male	21	20	
Female	7	8	
Tumor size			0.584
< 5 cm	18	16	
≥ 5 cm	10	12	
TNM stage			0.016*
I-II	19	10	
III-IV	9	18	
Lymph node metastasis			0.007*
Absent	17	7	
Present	11	21	
Histologic grade			
Low	14	11	0.420
Middle/High	14	17	

### Knockdown of circNTRK2 suppressed ESCC progression in vitro

**Fig. 2:**
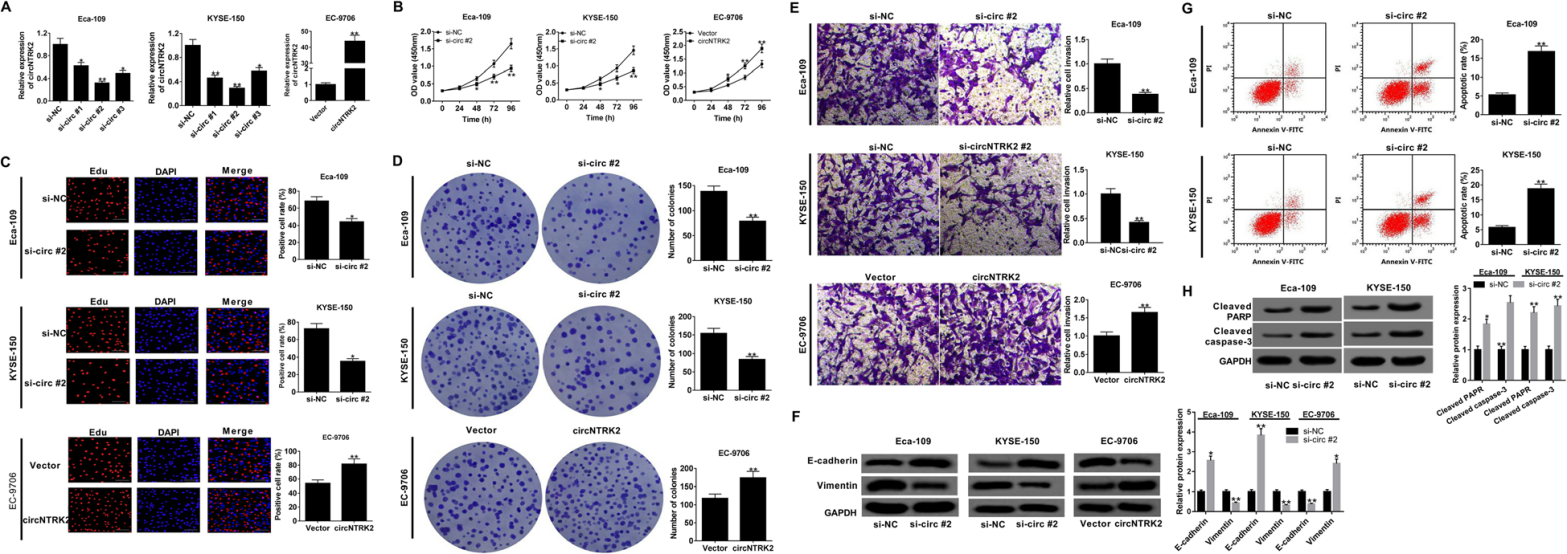
Effects of circNTRK2 on cell proliferation, invasion and apoptosis in ESCC. (**a**) Expression of circNTRK2 in Eca-109 and KYSE-150 cells transfected with si-RNAs (si-circNTRK2 #1, si-circNTRK2 #2, or si-circNTRK2 #3) and in EC-9706 cells transfected with circNTRK2-overexpression vector was tested by qRT-PCR. (**b**-**d**) Effects of circNTRK2 knockdown or overexpression on cell proliferation were monitored via CCK-8 (**b**), EdU (**c**), and colony formation (**d**) assays. (**e**) The invasive ability was assessed by transwell assay in circNTRK2-silencing Eca-109 and KYSE-150 cells and in circNTRK2-overexpression EC-9706 cells. (**f**) Western blot assay was performed to evaluate the effects of circNTRK2 depletion or up-regulation on the expression EMT-related proteins including E-cadherin and vimentin. (**g**) The apoptotic rate was detected through flow cytometry in Eca-109 and KYSE-150 cells transfected with si-circNTRK2 #2 and in EC-9706 cells transfected with circNTRK2. (**h**) Effects of circNTRK2 silencing or overexpression on the levels of apoptosis-related proteins (cleaved PARP and cleaved caspase-3) were measured via western blot assay. *P < 0.05, **P < 0.01

To explore the effects of circNTRK2 in ESCC progression, three different siRNAs targeting circNTRK2 (si-circNTRK2 #1, si-circNTRK2 #2, and si-circNTRK2 #3) were designed and transfected into Eca-109 and KYSE-150 cells, while circNTRK2-overexpressing vector (circNTRK2) was constructed and transfected into EC-9706 cells. As presented in Fig. 2A, circNTRK2 expression was significantly decreased in Eca-109 and KYSE-150 cells transfected with si-circNTRK2, while a dramatical up-regulation of circNTRK2 was found in circNTRK2-transfected group. Since si-circNTRK2 #2 possessed the highest knockdown efficiency, it was used in the following experiments. CCK-8 assay showed that down-regulation of circNTRK2 decreased cell viability in Eca-109 and KYSE-150 cells, while overexpression of circNTRK2 increased cell viability in EC-9706 cells (Fig. 2B). Similarly, EdU assays revealed that cell proliferation ability was notably inhibited in si-circNTRK2 group, but was apparently enhanced by circNTRK2 (Fig. 2C). Colony formation assay further verified the anti-proliferative roles of circNTRK2 knockdown in Eca-109 and KYSE-150 cells and the pro-proliferative effects of circNTRK2 up-regulation in EC-9706 cells (Fig. 2D). As demonstrated by transwell assay, silencing of circNTRK2 led to a decline of invasive ability in Eca-109 and KYSE-150 cells, while enforced expression of circNTRK2 resulted in an enhancement of invasion in EC-9706 cells (Fig. 2E). In agreement with the above findings, western blot clarified an increase of E-cadherin expression and a reduction of Vimentin expression in Eca-109 and KYSE-150 cells with circNTRK2 depletion, while circNTRK2 overexpression displayed an opposite effect (Fig. 2F). Flow cytometry analysis with Annexin V/PI double staining showed that down-regulation of circNTRK2 promoted apoptosis in Eca-109 and KYSE-150 cells (Fig. 2G). Consistently, knockdown of circNTRK2 in Eca-109 and KYSE-150 cells significantly enhanced the expression of apoptosis-related protein including cleaved PARP and cleaved caspase-3 (Fig. 2H). Taken together, these results indicated that knockdown of circNTRK2 inhibited ESCC cell proliferation and invasion, and induced apoptosis.

### CircNTRK2 served as a molecular sponge for miR-140-3p in ESCC cells

**Fig. 3:**
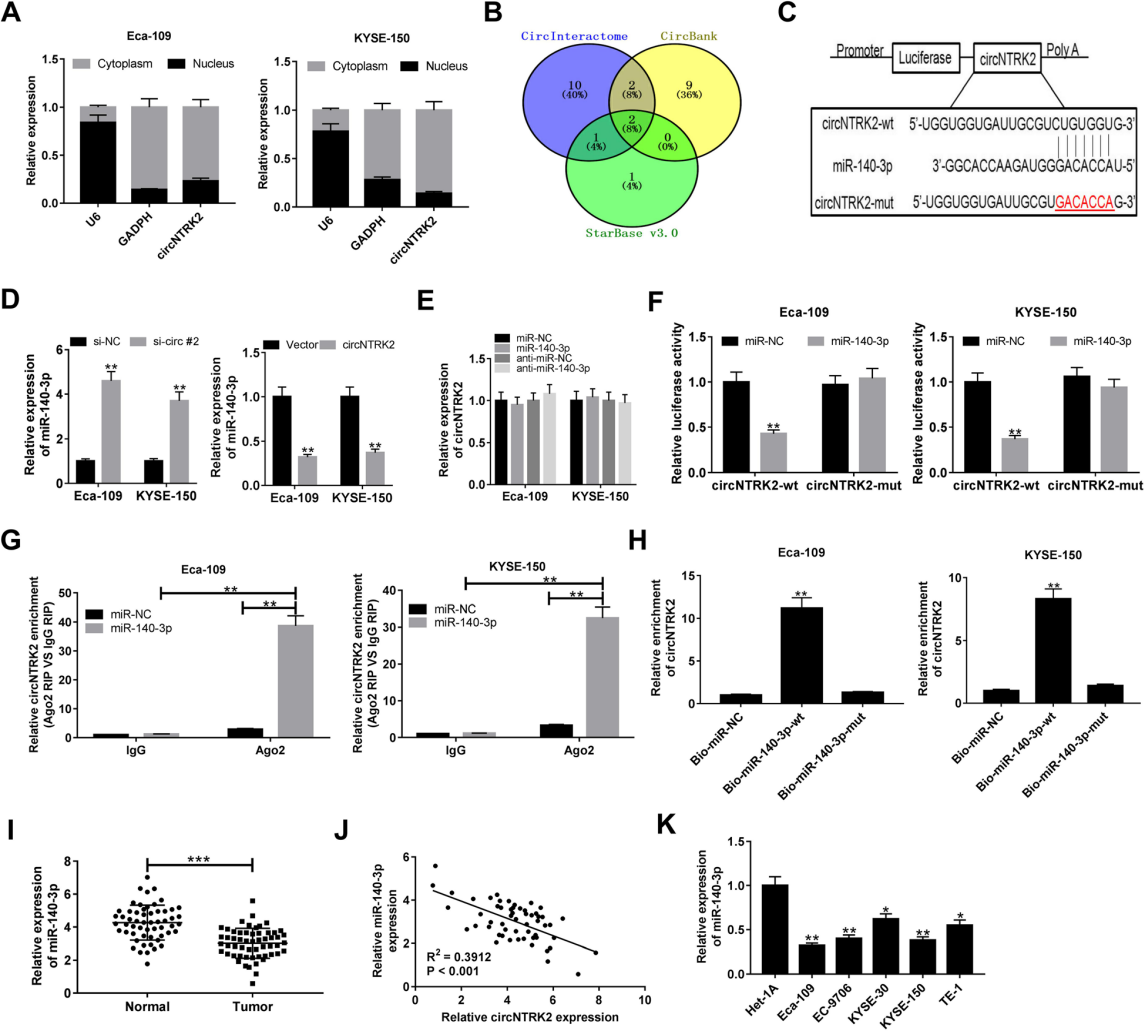
CircNTRK2 acts as a sponge for miR-140-3p of in ESCC cells. (**a**) The subcellular distribution of circNTRK2 in Eca-109 and KYSE-150 cells was determined by nuclear mass separation experiment. (**b**) Venn diagram showed the overlapping candidate targets of circNTRK2 predicted by CircInteractome, CircBank, and StarBase 3.0. (**c**) Schematic representation of miR-140-3p binding sites on circNTRK2. The red part is the mutated sequences of circNTRK2. (**d**) The level of miR-140-3p in Eca-109 and KYSE-150 cells transfected with si-circNTRK2 #2 or circNTRK2 was measured via qRT-PCR. (**e**) qRT-PCR was performed to evaluate the the expression of circNTRK2 in Eca-109 and KYSE-150 cells with miR-140-3p overexpression or depletion. (**f**) Dual-luciferase reporter assay was performed in Eca-109 and KYSE-150 cells after transfection with miR-NC or miR-140-3p and circNTRK2-wt or circNTRK2-mut reporter. (**g**) RIP assays were carried out in miR-NC- or miR-140-3p-transfected Eca-109 and KYSE-150 cells by using anti-Ago2 or anti-IgG, then qRT-PCR was used to examine the enrichment of circNTRK2 in immunoprecipitated RNA. (**h**) RNA pull-down assay was performed to confirm the direct binding between circNTRK2 and miR-140-3p. (**i**) The level of miR-140-3p in ESCC tissues was tested by qRT-PCR. (**j**) Correlation between miR-140-3p and circNTRK2 was detected in ESCC tissues via Pearson’s test. (**k**) Differential expression of circNTRK2 in ESCC cell lines (Eca-109, EC-9706, KYSE-30, KYSE-150, TE-1) and normal esophageal epithelial cell line Het-1A. *P < 0.05, **P < 0.01

In order to seek for the potential molecular mechanism of circNTRK2 in ESCC progression, subcellular fractionation was performed in ESCC cells. As exhibited in Fig. 3A, circNTRK2 was mostly located in the cytoplasm of Eca-109 and KYSE-150 cells. Accumulating evidence supports the idea that circRNAs could act as miRNA sponges to affect mRNA stability and transcription at the post-transcriptional level in cancer [16]. Thus, we speculate the oncogenicity of circNTRK2 in ESCC was attributed to the similar mechanism. Firstly, the putative candidate targets of circNTRK2 were searched in CircInteractome, CircBank, StarBase 3.0 online databases. The Venn diagram displayed hsa-miR-140-3p and hsa-miR-432-5p via overlapping the prediction results (Fig. 3B). According to the data from dbDEMC 2.0 (https://www.picb.ac.cn/dbDEMC/search.html), decreased expression of miR-140-3p was observed in esophagus cancer. Hence, miR-140-3p was chosen as the research subject. The complementary bases between circNTRK2 and miR-140-3p were shown in Fig. 3C. Moreover, silencing of circNTRK2 promoted the level of miR-140-3p, while overexpression of circNTRK2 reduced miR-140-3p expression in Eca-109 and KYSE-150 cells (Fig. 3D). Nevertheless, circNTRK2 expression was unchanged due to miR-140-3p up-regulation or knockdown (Fig. 3E). Subsequent dual-luciferase reporter assays showed that transfection of miR-140-3p lowered the luciferase activity of circNTRK2-wt reporter in comparison with that in miR-NC group; however, no significant fluctuation was found in the luciferase activity of circNTRK2-mut reporter between miR-NC and miR-140-3p group (Fig. 3F). RIP assay revealed that Ago2 pulled down much more circNTRK2 in miR-140-3p group than that in miR-NC group (Fig. 3G). RNA pull-down experiments clarified that in contrast to Bio-miR-NC, Bio-miR-140-3p-wt captured more circNTRK2 in ESCC cells, but the this effect was disappeared when the binding sites on circNTRK2 were mutated (Fig. 3H). As expected, miR-140-3p expression was confirmed to be lowered in ESCC tissue specimens and cell lines (Fig. 3I and K). Also, the scatter plot revealed that the level of miR-140-3p was negatively correlated with circNTRK2 expression in ESCC tissues (Fig. 3J). To sum up, circNTRK2 suppressed miR-140-3p expression in ESCC cells by acting as a molecular sponge.

### Transfection of circNTRK2 antagonized miR-140-3p-induced inhibitory effects on ESCC progression in vitro

**Fig. 4:**
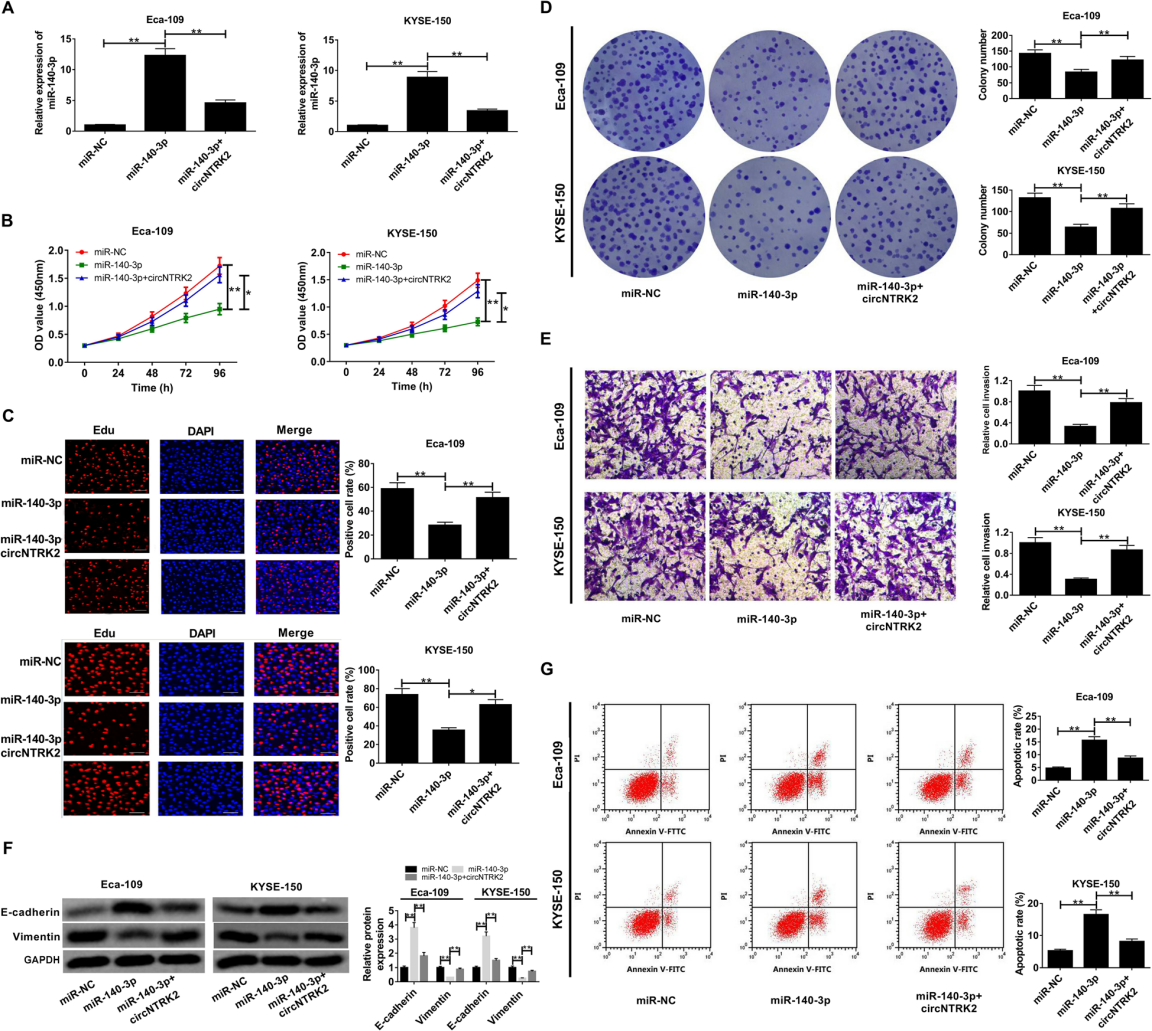
Enforced expression of circNTRK2 mitigated miR-140-3p-mediated inhibition of ESCC progression in vitro. (**a**-**g**) Eca-109 and KYSE-109 cells were transfected with miR-NC, miR-140-3p, or miR-140-3p + circNTRK2. (**a**) The miR-140-3p expression level was examined by qRT-PCR. (**b**) The cell viability was determined through CCK-8 assay. (**c**) The number of EdU-positive cell rate was detected by EdU assay. (**d**) Colony formation assay was performed to evaluate the colony-forming capability. (**e**) Transwell assay was used to measure the invasive ability of ESCC cells. (**f**) Western blot assay was used to measure the protein expression of E-cadherin and vimentin in ESCC cells. (**g**) Apoptosis analysis was conducted with Annexin V-FITC/PI double staining and flow cytometry. *P < 0.05, **P < 0.01

To gain insight into the functions of miR-140-3p, and further address whether it was associated with circNTRK2 in ESCC, Eca-109 and KYSE-150 cells were transfected with miR-140-3p alone or co-transfected with miR-140-3p and circNTRK2. As shown in Fig. 4A, the level of miR-140-3p was significantly up-regulated in Eca-109 and KYSE-109 cells transfected with miR-140-3p, while introduction of circNTRK2 reversed this promotion effect. Functionally, overexpression of miR-140-3p significantly repressed cell viability (Fig. 4B), EdU-positive cell rate (Fig. 4C) and colony forming ability (Fig. 4D), and such anti-proliferative effects were abated by the up-regulation of circNTRK2. Analogously, miR-140-3p mimic dramatically inhibited cell invasion, while this effect were neutralized after co-transfection with circNTRK2 (Fig. 4E). Moreover, E-cadherin expression was increased and Vimentin expression was declined in Eca-109 and KYSE-150 cells with miR-140-3p overexpression, however, these changes were greatly reversed after co-transfection with circNTRK2 and miR-140-3p (Fig. 4F). In addition, overexpression of miR-140-3p resulted in an increase of apoptotic rate in Eca-109 and KYSE-109 cells, whereas miR-140-3p-induced apoptosis was relieved following the up-regulation of circNTRK2 (Fig. 4G). Collectively, circNTRK2 promoted cell malignant phenotypes in ESCC through sponging miR-140-3p.

### CircNTRK2 acted as a sponge for miR-140-3p to facilitate NRIP1 expression in ESCC cells

**Fig. 5:**
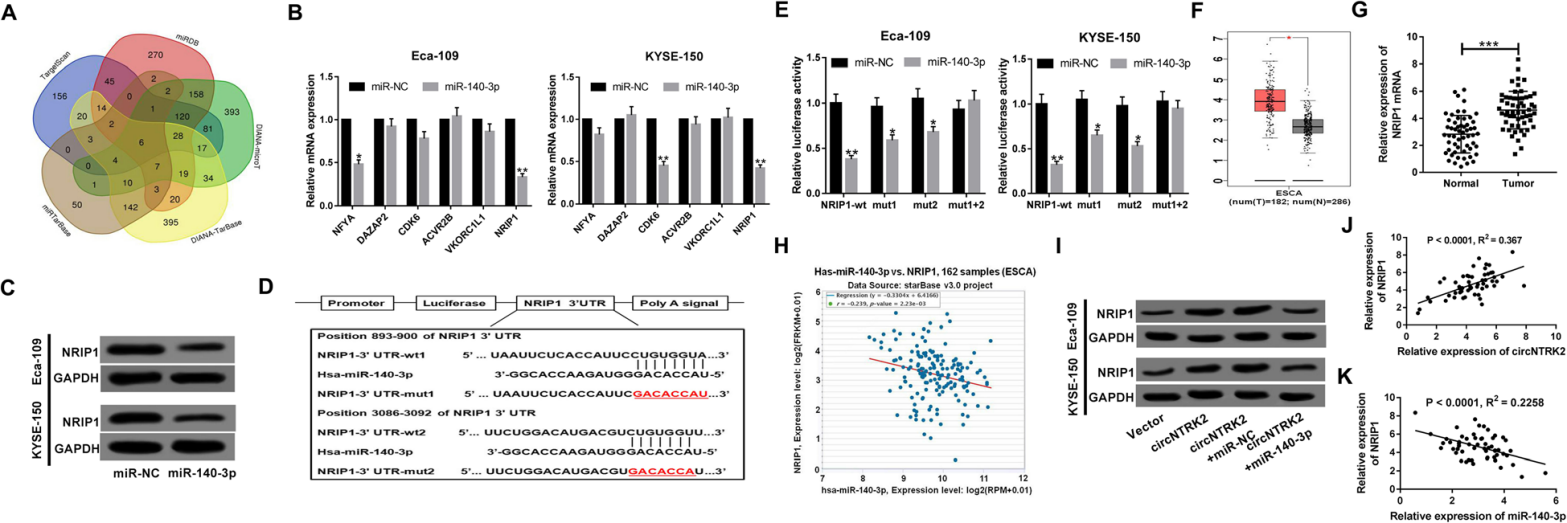
CircNTRK2 acted as a sponge for miR-140-3p to abate it suppression of NRIP1 in ESCC cells. (**a**) Venn diagram showing the overlap of putative targets of miR-140-3p predicted by TargetScan, miRDB, DIANA-microT, DIANA-TarBase, and miRTarBase online databases. (**b**) The levels of NFYA, DAZAP2, CDK6, ACVR2B, VKORC1L1, and NRIP1 in Eca-109 and KYSE-150 cells transfected with miR-140-3p or miR-NC were detected by qRT-PCR. (**c**) The effect of miR-140-3p on NRIP1 protein expression was tested via western blot assay in Eca-109 and KYSE-150 cells. (**d**) The complementary sequences between NRIP1–3’UTR and miR-140-3p was shown. The red section represents their mutant (mut1 and mut2). (**e**) The luciferase activity of NRIP-wt, −mut1, −mut2, or -mut1 + 2 reporter was measured by dual-luciferase reporter assay in Eca-109 and KYSE-150 cells after transfection with miR-140-3p or miR-NC. (**f**) Expression difference of NRIP1 in ESCC tissues and normal tissues were predicted by GEPIA tool. (**g**) The level of NRIP in 56 pairs of ESCC tumor tissues and adjacent non-cancerous was examined via qRT-PCR. (**h**) The correlation between NRIP1 and miR-140-3p expressions in esophageal cancer was predicted in starbase Pan-Cancer Analysis Platform. (**i**) The protein level of NRIP1 was measured by using western blot assay in Eca-109 and KYSE-150 cells after transfection with circNTRK2 or circNTRK2 + miR-140-3p. (**j** and **k**) The correlation between NRIP1 and circNTRK2 or miR-140-3p was analyzed by Pearson test. *P < 0.05, **P < 0.01, ***P < 0.001

To gain insight into the underlying mechanisms of miR-140-3p involved in ESCC, we used 5 online databases TargetScan, miRDB, DIANA-microT, DIANA-TarBase, and miRTarBase to search for the candidate targets of miR-140-3p. As presented in Venn Diagram, 6 putative targets (NFYA, DAZAP2, CDK6, ACVR2B, VKORC1L1, and NRIP1) were found (Fig. 5A). Then, the effects of miR-140-3p on these gene expressions were explored in Eca-109 and KYSE-150 cells. The result showed that only NRIP1 expression was declined in both Eca-109 and KYSE-150 cells transfected with miR-140-3p (Fig. 5B). Thus, NRIP1 was selected as the target gene for further experiments. Western blot assays revealed that overexpression of miR-140-3p repressed the protein level of NRIP1 in Eca-109 and KYSE-150 cells (Fig. 5C). As exhibited in Fig. 5D, the complementary binding sites between 3’UTR of NTRP1 and miR-140-3p were predicted in two sites (893–900 and 3086–3092). Dual-luciferase reporter assay demonstrated that miR-140-3p mimic significantly decreased the luciferase activity of NRIP1-wt reporter in Eca-109 and KYSE-150 cells. Single mutation of the putative binding sequences in NRIP1-wt, namely NRIP1-mut1 or NRIP1-mut2, partially restored the luciferase activity suppressed by miR-140-3p, while mut1 + 2 reporter with double mutation completely reversed miR-140-3p-induced inhibition of luciferase activity (Fig. 5E). According to Gene Expression Profiling Interactive Analysis (GEIPA) database, level of NRIP1 was higher in tumor tissues than that in normal specimens in esophageal carcinoma (Fig. 5F). Subsequent qRT-PCR results further verified the up-regulation of NRIP1 in ESCC tissues when compared with their adjacent normal tissues (Fig. 5G). Besides, a negative correlation between NRIP1 and miR-140-3p was observed in esophageal carcinoma by using starBase Pan-Cancer Analysis (Fig. 5H). As depicted by western blot assay in Fig. 5I, the protein level of NRIP1 was elevated in circNTRK2-transfected Eca-109 and KYSE-150 cells, while this effect was attenuated by the recovery of miR-140-3p expression. The scatter diagram presented that NRIP1 expression was positively correlated with circNTRK2, but negatively associated with miR-140-3p in ESCC tissues (Fig. 5J and K). These findings supported that circNTRK2 up-regulated NRIP1 expression in ESCC cells through sponging miR-140-3p.

### Depletion of circNTRK2 repressed cell malignant behaviors in ESCC by regulating NRIP1

**Fig. 6:**
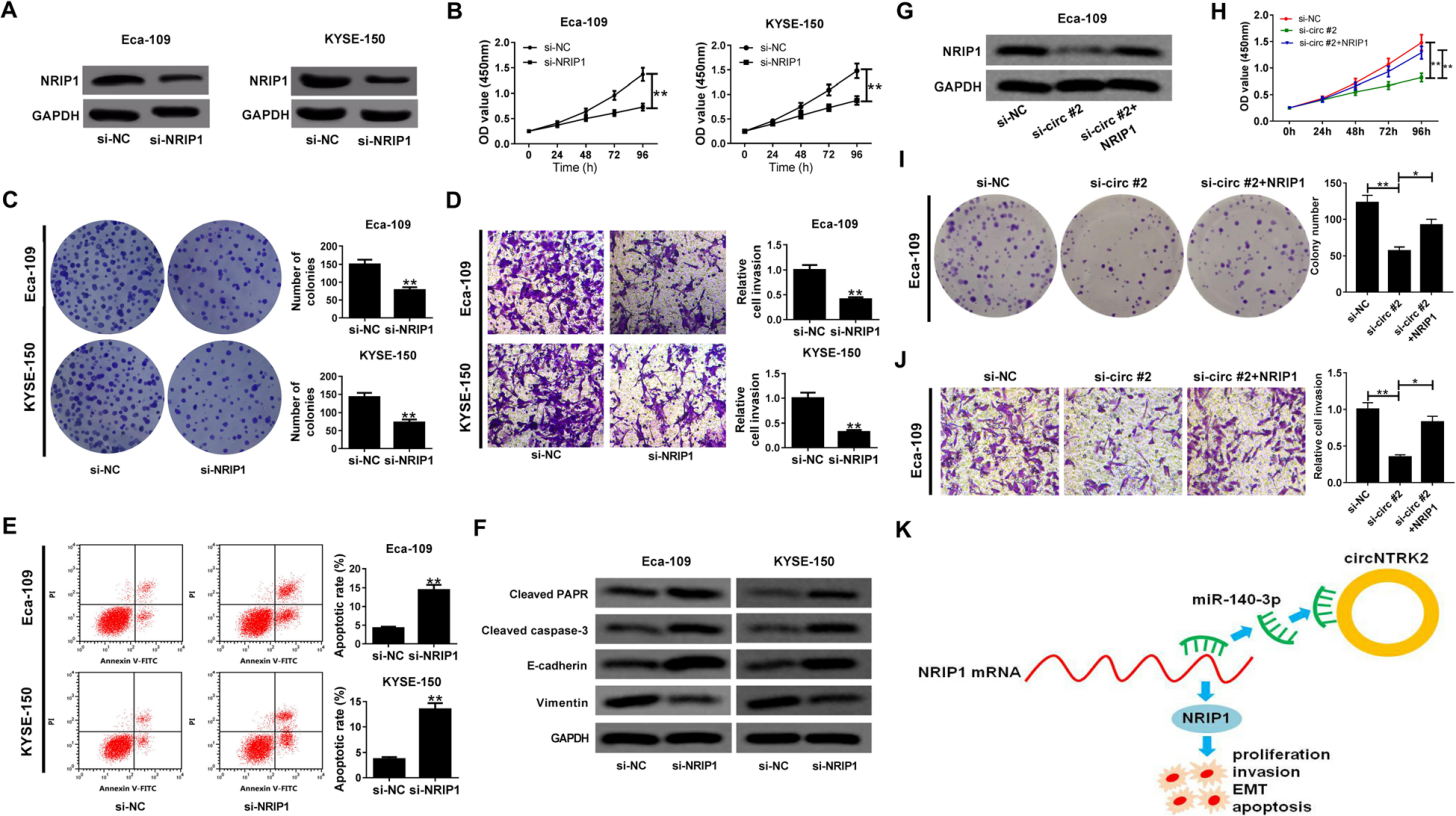
CircNTRK2 promotes ESCC cell malignant behaviors by modulating NRIP1 expression. (**a**) The protein level of NRIP1 in si-NRIP1-transfected Eca-109 and KYSE-150 cells was determined by western blot assay. (**b**) The effect of NRIP1 knockdown on cell viability was assessed by CCK-8 assay. (**c**) Colony formation assay in Eca-109 and KYSE-150 cells with NRIP1 depletion. (**d**) Transwell assay was used to evaluate the effect of NRIP1 down-regulation on cell invasive capacity. (**e**) Flow cytometry was performed to measure the apoptotic rate of Eca-109 and KYSE-150 cells transfected with si-NRIP1. (**f**) The protein levels of cleaved PARP, cleaved caspase-3, E-cadherin and vimentin were examined by western blot assay in Eca-109 and KYSE-150 cells. (**g**-**j**) Eca-109 cells were transfected with si-NC, si-circ #2 or si-circ #2 + NRIP1, followed by (**g**) western blot assay to evaluate the protein level of NRIP1; (**h**) CCK-8 assay to determine cell viability; (**i**) colony forming assay to detect colony-formation ability; (**j**) transwell assay to examine cell invasiveness. (**k**) The schematic diagram shows that circNTRK2 acts as a sponge for miR-140-3p to relieve its suppression on NRIP1 expression, thereby contributing to ESCC proliferation and metastasis. *P < 0.05, **P < 0.01

Next, a specific siRNA targeting NRIP1 (si-NRIP1) was transfected into ESCC cells to investigate the function of NRIP1 in ESCC. The knockdown efficiency was confirmed by western blot assay in Eca-109 and KYSE-150 cells (Fig. 6A). Functionally, knockdown of si-NRIP1 resulted in a striking suppression of cell viability (Fig. 6B), colony formation ability (Fig. 6C) and invasiveness (Fig. 6D). Moreover, cell apoptosis was greatly increased in Eca-109 and KYSE-150 cells with NRIP1 down-regulation (Fig. 6E). Consistently, silencing of NRIP1 lead to an increase of cleaved PARP, cleaved caspase-3 and E-cadherin expression, while a decrease of Vimentin expression (Fig. 6F). These data indicated that knockdown of NRIP1 exerted a tumor-suppressive effect in ESCC in vitro.

To further confirm the detailed mechanism of circNTRK2 in ESCC, rescue experiments were performed in Eca-109 cells by transfection with si-circ #2 or si-circ #2 + NRIP1. As shown in Fig. 6G, si-circ #2-induced suppression of NRIP1 was reversed by co-transfection with NRIP1-overexpression vector. Moreover, si-circ #2-mediated inhibition of cell proliferation, colony forming ability and invasion was markedly abated by NRIP1 up-regulation (Fig. 6H-J). By and large, circNTRK2 facilitated ESCC progression through regulating NRIP1 expression.

### Silencing of circNTRK2 hindered xenograft tumor growth in vivo

**Fig. 7:**
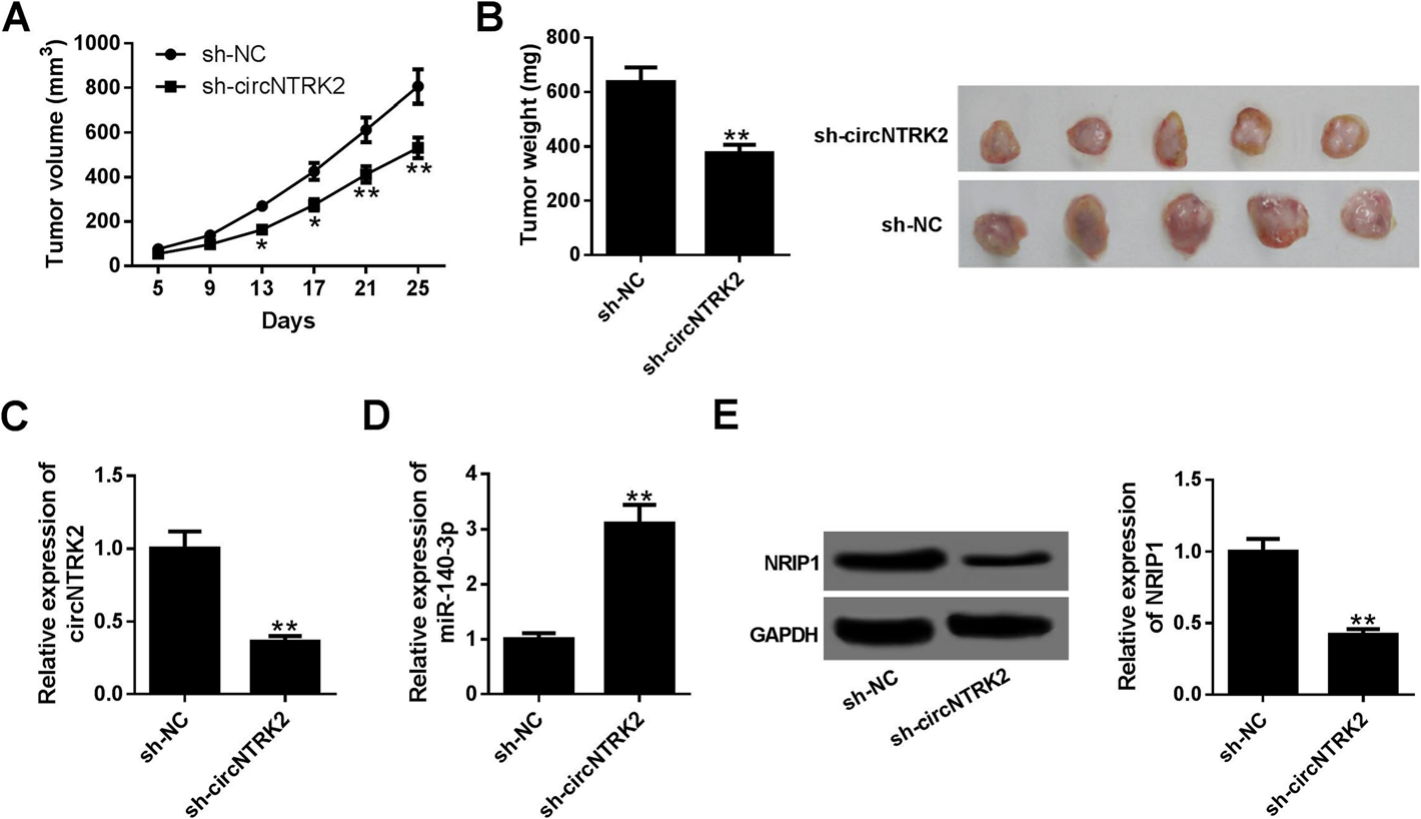
CircNTRK2 silencing inhibited tumor formation in xenografted nude mice*.* (**a**-**e**) KYSE-150 cells stably transfected with sh-circNTRK2 were implanted into nude mice. (**a**) The growth curve of xenograft tumors was shown. (**b**) Tumor weight measurement in sh-NC- or sh-circNTRK2-treated nude mice, and representative images of excised tumor masses. (**c**-**d**) The levels of circNTRK2 and miR-140-3p were examined in tumors via qRT-PCR. (**e**) The protein levels of NRIP1 were tested in xenografts by western blot assay. *P < 0.05, **P < 0.01

To validate the effects of circNTRK2 on ESCC progression in vitro, KYSE-150 cells stably infected with lentivirus vectors encoding sh-circNTRK2 or sh-NC were injected into nude mice. As depicted in Fig. 7A, tumor growth was significantly slowed down by the knockdown of circNTRK2. Consistently, the weights of tumors derived from sh-circNTRK2-transfected cells were decreased compared to the control group (Fig. 7B). As we might expect, the level of circNTRK2 were declined, while miR-140-3p expression was increased in the excised tumor tissues of sh-circNTRK2 group compared to that of sh-NC group (Fig. 7C and D). Moreover, silencing of circNTRK2 led to a down-regulation of NRIP1 protein level (Fig. 7E). These data demonstrated that depletion of circNTRK2 impeded ESCC tumor growth in vivo.

## Discussion

Although there is a slight decline in the global incidence of ESCC in recent years, it is still a primary cause of cancer-related mortality worldwide [17]. CircRNAs have drawn increasing attentions for their important roles in the initiation and progression of human cancers [18]. However, much is still undiscovered about the precise roles of circRNAs in ESCC. A deeper understanding of the mechanisms of circRNAs is vital to discover the promising biomarkers and targets for ESCC patients. Based on the information from GEO database (GSE131969), we selected circNTRK2 to elucidate its biological significance and underlying mechanisms in ESCC. Our results demonstrated that circNTRK2 served as a sponge for miR-140-3p to relieve its inhibition on NRIP1, thus contributing to cell proliferation and invasion in ESCC.

Up to now, increasing circRNAs have been discovered to be associated with the pathophysiological events in ESCC. For example, hsa_circ_0006948 was up-regulated in ESCC, and induced HMGA2 expression to facilitate ESCC progression via miR-490-3p [19]. Hsa-circ_0000654 expression was increased in ESCC tissues, and knockdown of circ_0000654 repressed cell growth and metastasis through miR-149-5p/STAT3 axis [20]. Circular RNA ciRS-7 promoted ESCC growth and metastasis via serving as a miR-876-5p sponge to increase MAGE-A family expression [21]. In the current study, circNTRK2 was confirmed as a circular RNA through Sanger sequencing, PCR and RNase R treatment. CircNTRK2 expression was elevated in ESCC tissues and cells. Moreover, high circNTRK2 expression was associated with advanced TNM stage, lymph node metastasis and poor prognosis. Knockdown of circNTRK2 inhibited ESCC cell proliferation, invasion and EMT, and enhanced apoptosis, while overexpression of circNTRK2 displayed the contrary effect. These data suggested the carcinogenicity of circNTRK2 in ESCC. However, another study showed that hsa_circ_0087378 (circNTRK2) was down-regulated in tumor tissues and cell lines in ER-positive BC, and hsa_circ_0087378/miR-1260b/SFRP1 was concluded as its possible regulatory mechanism [15]. The controversy may be attributed to the cell-type specific features of circular RNA expression [22].

In recent years, circRNAs are known as competing endogenous RNA (ceRNA) to influence miRNAs stability and expression, thereby alleviating their inhibition of target genes [23]. By using subcellular fractionation assay, circNTRK2 was found to predominantly exist in the cytoplasm, implying that it may exert effect through post-transcriptional regulation. Hence, we speculated that circNTRK2 was involved in the regulation of ESCC through the similar ceRNA mechanism. On the basis of the prediction from bioinformatic tools and the data from luciferase reporter, RIP and RNA pull-down assays, miR-140-3p was confirmed as a direct target of circNTRK2. MiR-140-3p was previously demonstrated as a tumor-suppressor in some types of human malignancies, such as squamous cell lung cancer [24], breast cancer [25], hepatocellular carcinoma [26] and cervical cancer [27]. In the present study, miR-140-3p expression was down-regulated in ESCC tissues and cells, and was inversely correlated to circNTRK2 expression. Functionally, overexpression of miR-140-3p repressed cell proliferation and invasion, and promoted apoptosis, suggesting the anti-tumor effect of miR-140-3p in ESCC. However, miR-140-3p-induced suppression of cell proliferation and invasion was evidently reversed following the introduction of circNTRK2. From the above results, we concluded that circNTRK2 accelerated ESCC progression via sponging miR-140-3p.

Nuclear receptor-interacting protein 1 (NRIP1), also known as RIP140, was originally identified in breast cancer cells through its interaction with the estrogen receptor α [28]. NRIP1 is associated with the regulation of various oncogenic signaling pathways and participates in the progression of solid tumors [29]. For instance, down-regulation of NRIP1 by siRNA inhibited breast cancer cell growth in vitro and in vivo [30]. NRIP1 was demonstrated as an independent predictor of poor survival for cervical cancer patients [31]. However, NRIP1 was found as a tumor-suppressor in some other malignancies, such as nasopharyngeal carcinoma [32], hepatocellular carcinoma [33], and colon cancer [34]. In our study, NRIP1 was verified as a target of miR-140-3p in ESCC cells, and NRIP1 was highly expressed in ESCC tissues. Moreover, circNTRK2 overexpression led to an increase of NRIP1, while this effect was attenuated by the restoration of miR-140-3p. Furthermore, NRIP1 expression was positively associated with circNTRK2, while was negatively correlated to miR-140-3p in ESCC tissues. Thus, a conclusion was reached that circNTRK2 functioned as a miR-140-3p sponge to abolish its inhibition of NRIP1. Loss-of-function experiments revealed that silencing of NRIP1 suppressed cell proliferation and invasion, but facilitated apoptosis in ESCC. Moreover, the anti-proliferation and anti-invasion effects induced by circNTRK2 knockdown were greatly abrogated by the overexpression of NRIP1. To sum up, circNTRK2 promoted ESCC progression by sponging miR-140-3p and stimulating NRIP1. Our study elucidated a circNTRK2-miR-140-3p-NRIP1 regulatory axis in ESCC (Fig. 6K). In addition to the “miRNA sponges” function, circRNAs can also interact with RNA binding proteins, regulate modulate mRNAs stability, modulate gene transcription, and act as translation templates of proteins [18, 35]. The other possible action mechanisms of circNTRK2 in ESCC will be further investigated in our future research.

## Conclusion

In summary, circNTRK2 expression was increased in ESCC tissues and cells. High circNTRK2 expression was correlated with advanced TNM stage, lymph node metastasis and poor prognosis. Further functional and mechanistic experiments unraveled that circNTRK2 modulated NRIP1 expression by sponging miR-140-3p, contributing to the malignant cell behaviors of ESCC. Our findings provide a theoretical guidance for understanding the molecular mechanism of circNTRK2 and elucidate a potential therapeutic target in ESCC. However, due to the complexity of circNTRK2 regulatory networks as a ceRNA, more downstream targets and related signaling pathways are needed to be further investigated.

## Supplementary information

**Additional file 1 **Fig. 1**.** Expression of circNTRK2 in 35 pairs of ESCC tumor tissues and adjacent non-cancerous tissues was measured by qRT-PCR.

## Data Availability

The datasets used and analysed during the current study are available from the corresponding author on reasonable request.

## Abbreviations

ESCC: Esophageal squamous cell carcinoma
circRNAs: Circular RNAs
NRIP1: Nuclear receptor-interacting protein 1
qRT-PCR: Quantitative real-time polymerase chain reaction
cDNA: Complementary DNA
gDNA: Genomic DNA
siRNA: Small interfering RNA
CCK-8: Cell counting kit-8
EdU: 5-Ethynyl-2′- deoxyuridine
RIP: RNA immunoprecipitation
GEIPA: Gene Expression Profiling Interactive Analysis
SDS-PAGE: Sodium dodecyl sulfonate-polyacrylamide gel electrophoresis
PVDF: Polyvinylidene fluoride
