# Supplementary figures and images for: Circular RNA circNTRK2 facilitates the progression of esophageal squamous cell carcinoma through up-regulating NRIP1 expression via miR-140-3p
Source: J Exp Clin Cancer Res. 2020 Jul 11;39:133. doi: 10.1186/s13046-020-01640-9 (PMC7353745; doi:10.1186/s13046-020-01640-9)

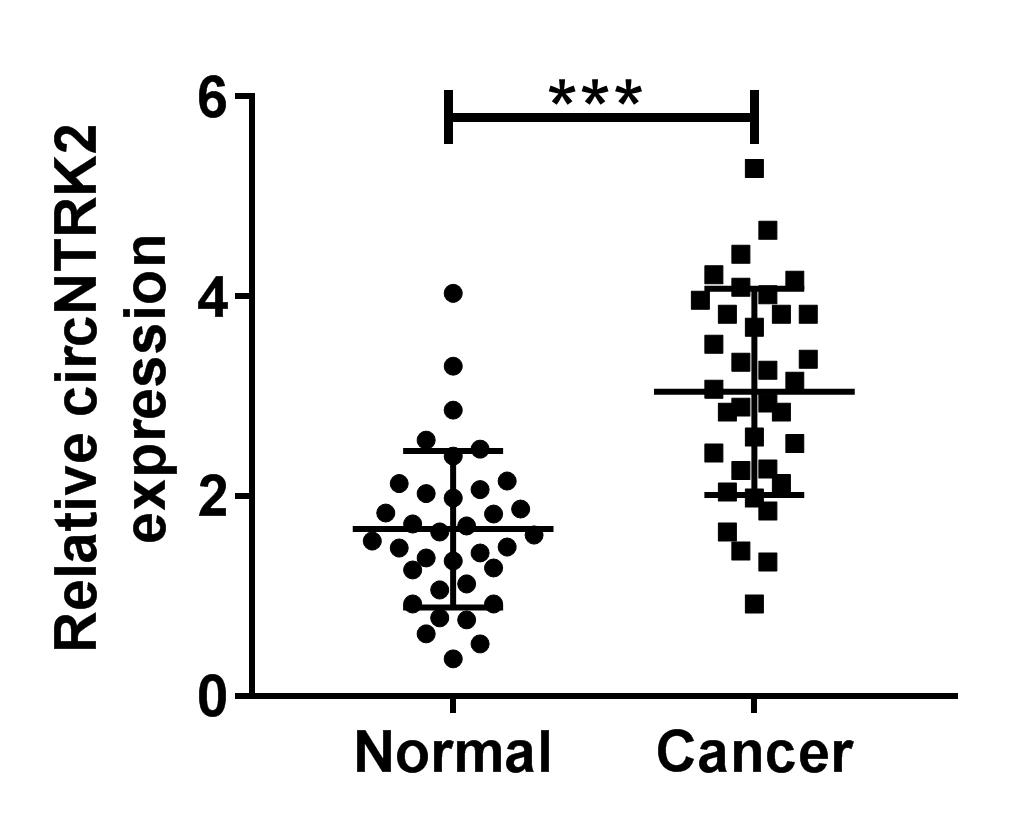

Supplement: Supplementary file 1 — Additional file 1 Fig. 1. Expression of circNTRK2 in 35 pairs of ESCC tumor tissues and adjacent non-cancerous tissues was measured by qRT-PCR. [file 13046_2020_1640_MOESM1_ESM.jpg]
